# Solvent-Impregnated Resins Based on the Mixture of (2-Diphenylphosphoryl)-4-ethylphenoxy)methyl) diphenylphosphine Oxide and Ionic Liquid for Nd(III) Recovery from Nitric Acid Media

**DOI:** 10.3390/molecules26092440

**Published:** 2021-04-22

**Authors:** Olga Kovalenko, Vladimir Baulin, Dmitriy Baulin, Aslan Tsivadze

**Affiliations:** 1 A. N. Frumkin Institute of Physical Chemistry and Electrochemistry, Russian Academy of Sciences, Leninsky Prospect 31, Building 4, 119071 Moscow, Russia; olga_smit@mail.ru (O.K.); atsiv43@mail.ru (A.T.); 2 Institute of Physiologically Active Substances, Russian Academy of Sciences, Severnyi Proezd 1, Chernogolovka, 142432 Moscow Region, Russia; mager1988@gmail.com

**Keywords:** phosphorylpodands, ionic liquid, solvent impregnated resins, synergistic effect, extraction chromatography, Nd(III)

## Abstract

Novel solvent-impregnated resins (SIRs) were prepared by treatment of styrene–divinylbenzene copolymer (LPS-500) with mixtures of the promising polydentante extractant (2-diphenylphosphoryl)-4-ethylphenoxy)methyl)diphenylphosphine oxide (L) and an ionic liquid [C_4_mim]^+^[Tf_2_N]^−^for the extraction chromatography recovery of Nd(III) from nitric acid solutions. It was shown that introduction of the ionic liquid into the SIR composition results in considerable enhancement of the Nd(III) recovery efficiency compared with resin impregnated only by L in slightly acidic media. The influence of the L: ionic liquid molar ratio in the SIRs composition, their percentages, concentration of metal and HNO_3_ in the eluent, and acid type on the value of synergistic effect and adsorption efficiency of Nd(III) recovery was studied. The SIR containing 40% of mixture of L and [C_4_mim]^+^[Tf_2_N]^−^ with molar ratio 2:1 turned out to be the most efficient. The selectivity of Nd(III) separation from light and heavy rare-earth elements was studied and the optimal conditions of Nd(III) adsorption recovery and stripping by this SIR were chosen. It was found that in recovery efficiency of Nd(III) developed SIR exceeded the SIR containing Cyanex 923 (a mixture of monodentate trialkylphosphine oxides) and [C_4_mim]^+^[Tf_2_N]^−^.

## 1. Introduction

Applications of rare-earth elements (REEs) in various high technology industries are conditioned by their unique physical properties. Satisfying the needs of various industries, such as metallurgy, nuclear energy, manufacture of optical, magnetic, luminescent, and laser materials, and petrochemicals most often requires high purity individual REEs. Among the rare earth elements neodymium is important for doping alloys [1] and manufacturing permanent magnets [2], laser production [3], and preparing catalysts [4,5]. Due to their similar physical and chemical properties the recovery of individual rare earth metals is a complex and yet partially unresolved problem [6]. The most important REEs minerals processed in industrial scale are monazite, bastnesite, and xenotime [7]. However, due to the growing global demand for REEs their recovery from alternative secondary resources such as phosphate ores [8], coal combustion products [9], permanent magnets [10], mine tailings [11], etc. has become more relevant. At the same time, the need to extract REE from secondary resources gives rise to new scientific challenges for the development of new effective approaches to the extraction of REE due to the complex multicomponent composition of these resources and the extremely low content of REE in them [12].

Currently, extraction chromatography methods based on the use of highly selective solvent-impregnated resins (SIRs) are becoming increasingly important for solving problems of concentration, recovery, and separation of elements with similar properties [13]. Extraction chromatography combines the efficiency of liquid-liquid extraction with the simplicity and convenience of performing adsorption methods [14]. However, for the successful implementation of the extraction chromatography process, it is necessary to develop new extraction chromatographic materials.

Synthetically available SIRs in which a selective organic ligand (extractant) is non-covalently fixed on the surface of an inert support are most widely used in extraction chromatography [15]. An important aspect of the development of effective SIRs is the optimization of their stationary phase composition, which includes both the choice of a selective organic extractant and a suitable diluent. The latter can affect both the efficiency of metal recovery (values of distribution coefficients and resin capacity), and the selectivity of metal separation (values of metal separation factors) [16,17]. The choice of organic extractants and diluents for preparation of SIRs is commonly performed by using the results of preliminary experiments of liquid–liquid extraction.

For REEs liquid-liquid extraction and separation alkyl-substituted phosphorus acids are used such as di-(2-ethylhexyl)phosphoric acid (DEHPA) [18], 2-ethylhexylphosphonic acid mono-2-ethylhexyl ester (PC88A) [19], bis-(2,4,4-trimethylpentyl)phosphinic acid (Cyanex 272) [20], as well as neutral organophosphorus extractants, for example, tributylphosphate (TBP) [21], trialkylphosphine oxides (Cyanex 923) [22], dicarboxylic acid amides such as N,N-dioctyldiglycolamic acid (DODGAA) [23], and N,N,N′,N′-tetraoctyldiglycolamide (TODGA) [8],. However, these extractants do not always allow solving the problem of selective separation of individual REEs. 

The development of organic synthesis has led to the creation of new synthetically available polydentate extractants such as phosphorylpodands of neutral [24,25,26,27], and acidic type [28,29,30]. They possess high extraction ability and selectivity in relation to a wide range of chemical elements. Varying of substituents at the phosphoryl group and the design of polyether chain are effective approaches toward obtaining of phosphorylpodands with suitable extraction properties in relation to various metals, REEs inclusively. In a review [31] the applications of series of phosphorylpodands as extractants in impregnated resins for processing radioactive waste are reported.

**Figure 1:**
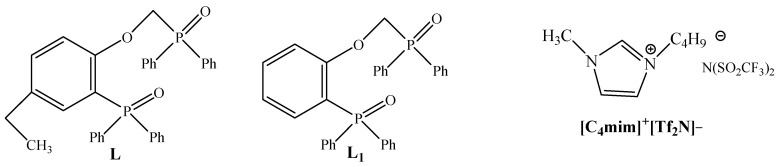
Structural formulas of the studied compounds.

Short-chain phosphorylpodand (2-diphenylphosphoryl-4-ethylphenoxy)-methyl)diphenylphosphine oxide (L) (Figure 1) is a promissing extractant for REEs recovery from nitric acid solution. 

In our previous report, we studied the effect of substituents at phosphoryl groups of compound L on the efficiency of liquid-liquid extraction of REEs [32]. Additionally, extraction of REEs, U(VI), and Th(IV) from perchlorate solutions into dichloroethane was investigated. The stoichiometry of the extractable complexes was determined, and the influence of the aqueous phase composition on the efficiency and selectivity of the extraction of U(VI), Th(IV), and REEs into the organic phase was explored [33]. The solution of L in 1,1,7-trihydrododecafluoroheptanol have also been investigated for rare earth element extraction from nitric acid media. It was shown that the values of distribution coefficients of REEs are negligible at nitric acid concentration lower 1 mol/L. Distribution coefficient sharply increases with nitric acid concentration from 1 mol/L and reaches 5.5 for the yttrium subgroup elements at HNO_3_ concentration of 6 mol/L. The rare earth elements of the yttrium subgroup were found to be extracted much better than the rare earth elements of the cerium one under the same conditions. Additionally, the values of distribution coefficients in both subgroups smoothly rise with atomic number of element. It was established using the method of extraction equilibrium shift that the metal: L ratio in extracted complexes is 1:2 irrespective of the nature of the rare earth element. The structure of the complex of Yb with L was determined by an X-ray diffraction study [34]. The complex of L with neodymium was studied by X-ray structural analysis and IR spectroscopy [35]. 

Recently increasing attention is focused on using of ionic liquids as promising dilutents both in liquid–liquid extraction [36,37,38], and as components of SIRs [39,40,41,42]. In both cases, the using of ionic liquids leads to a significant improvement of REEs recovery efficiency. Unlike traditional organic solvents, ionic liquids are not flammable or toxic, and have low pressure of vapor and high electric conductivity [43]. In the composition of SIRs ionic liquids may be used both independently [42,44] and as mixture with different extractants [41,45,46,47]. In the present report we are interested in the second case, that’s why first we consider extraction of REEs by such systems.

Numerous reports on the liquid–liquid extraction of REEs with different neutral extractants in the presence of variously structured ionic liquids have shown that the higher the hydrophilicity of the cation and the hydrophobicity of the anion in ionic liquids, the greater is the extraction of REEs [38,48,49,50]. Varying cations and anions of various structures yields ionic liquids with suitable properties necessary for certain application [43,51,52]. An important feature of ionic liquids is that their components can serve as hydrophobic counterions in the extracted metal complexes during their recovery from aqueous phase by various extractants -[36]. The most pronounced improvement in the efficiency of REE recovery in liquid-liquid extraction was achieved in the presence of ionic liquids of bis [(trifluoromethyl) sulfonyl] imide-1-alkyl-3-methylimidazolium derivatives ([C_n_mim]^+^[Tf_2_N]^−^ (where *n* = 4, 6, 8))]. 

Recently, we have found that liquid-liquid extraction of REEs with solutions of some neutral phosphorylpodands in dichloroethane from nitric acid media significantly increases in the presence of [C_4_mim]^+^[Tf_2_N]^−^ [53,54,55]. 

In this report, novel SIRs containing (2-diphenylphosphoryl)-4-ethylphenoxy)methyl) diphenylphosphine oxide and ionic liquid [C_4_mim]^+^[Tf_2_N]^−^ were obtained and the features of the chromatographic extraction of Nd (III) from nitric acid solutions were studied as well. The selectivity of Nd(III) recovery at the presence of La(III), Dy(III), and Tm(III) was examined. The comparison of recovery efficiency of Nd(III) by novel SIR and resin based on the mixture of Cyanex 923 (a mixture of monodentate trialkylphosphine oxides) and [C_4_mim]^+^[Tf_2_N]^−^ was performed.

## 2. Results

### 2.1. The Influence of Various Factors on Adsorption Recovery of Nd(III)

#### 2.1.1. The Influence of HNO_3_ Concentration

The influence of HNO_3_ concentration on the adsorption recovery of Nd(III) with SIRs impregnated by phosphorylpodand L alone (SIR 10), [C_4_mim]^+^[Tf_2_N]^−^ alone (SIR 11), and their mixture (SIR 4) was studied. The values of distribution coefficient (K_d_) of Nd(III) for these SIRs were established at the same conditions with varied values of HNO_3_ concentration in the eluent while the percentage of extractants in SIRs and the mass of SIRs in column were identical. 

**Figure 2:**
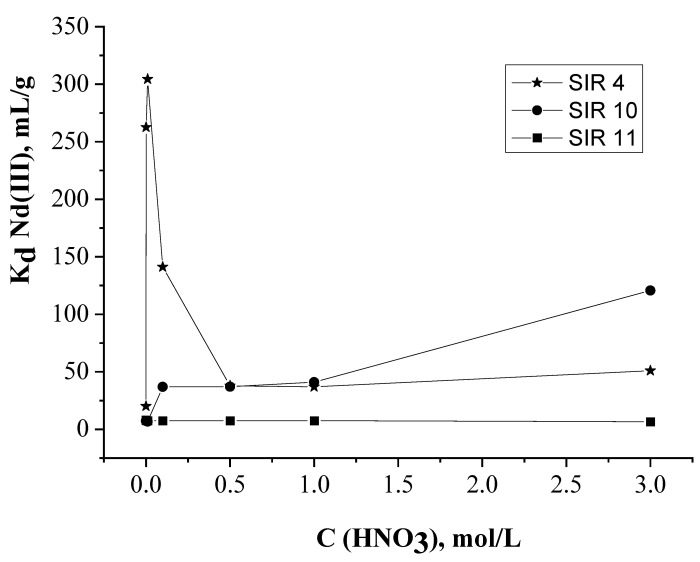
The influence of HNO_3_ concentration on the value of distribution coefficient of Nd(III).

SIR 4 extracts Nd(III) much better than SIR 10 and SIR 11 in the range of HNO_3_ concentrations from 0.001 to 0.1 (Figure 2). This means that introduction of an ionic liquid into the SIR composition results in a synergistic enhancement of the value of Nd(III) K_d_ when it is extracted by SIR 4 in slightly acidic media, which is likely due to the replacement of NO_3_^-^ by more hydrophobic Tf_2_N^-^ in extracted complexes of Nd(III) [50]. It should be noted that the recovery of Nd(III) by SIR 4 is the best in slightly acidic media, which allows to avoid the equipment corrosion. Moreover, in this case there is no need to use the salting-out agents to achieve quantitative recovery of Nd(III) in contrast to SIR 10 [56]. When the concentration of HNO_3_ is more than 0.01 mol/L the values of Nd(III) K_d_ decrease, likely due to the decline of concentration of free compound L due to its protonation.

Recently, we have studied the liquid-liquid extraction of REEs by mixture of (2-((diphenylphosphoryl)methoxy)phenyl)diphenylphosphine oxide L_1_ (a structural analog of L, Figure 1) and ionic liquid [C_4_mim]^+^[Tf_2_N]^−^ from nitric acid solutions [33]. It was established that the REE:L_1_ stoichiometric ratio in the extracted complexes in the presence of [C_4_mim]^+^[Tf_2_N]^−^ varied from 1:3 to 1:2 as the HNO_3_ concentration in the aqueous phase increases, i.e., the system with ionic liquid displays a solvation number growth in extracted complexes as compared with REEs extraction by solutions of phosphorylpodand in dichloroethane. REEs are extracted with L_1_ as mixture of mono- and disolvates in presence of dichloroethane [33].The mechanism of REEs liquid-liquid extraction with neutral polydentate extractants in the presence of ionic liquids is considered in detail in [37,38]. The extraction of REEs by mixture of neutral organophosphorus extractant (E) and ionic liquid [C_n_mim]^+^Tf_2_N^−^ can be described using Equation (1) [37,54]:

Ln^3+^_(a)_ + sE_(o)_ + [C_n_mim]^+^[Tf_2_N]^−^_(o)_ = LnE_s_(Tf_2_N)_3(o)_ + 3[C_n_mim]^+^_(a)_ (1)

where “s” is the solvation number; (a) and (o) denote components of aqueous and organic phases, respectively. From Equation (1) it is seen that extraction of REEs by organophosphorus extractants in presence of ionic liquid can be achieved via cation exchange mechanism which is accompanied with transfer of [C_n_mim]^+^ to the aqueous phase. Lower concentrations of free L in the organic phase due to its interaction both with HNO_3_, and HTf_2_N presented in system are due to an marked transfer of C_4_mim^+^ and Tf_2_N^-^ into the aqueous phase seem to account for lower K_d_ of Nd(III) with HNO_3_ concentration over 0.01 mol/L [54]. 

The dependence of Nd(III) K_d_ from HNO_3_ concentration for SIR 4 is substantially different from that observed for SIR 10. For the latter case, the increase of K_d_ along with the increase of HNO_3_ concentration is typical for neutral extractants, which was noted in numerous reports [57,58,59]. When the concentration of HNO_3_ is over 1 mol/L, Nd(III) is recovered by SIR 10 much better than by SIR 4, which should be attributed to the salting-out effect of NO_3_^−^] [60,61] that provides for rise values of Nd(III) K_d_ for SIR 10, but hardly influences the Nd(III) recovery by SIR 4. The latter is in accordance with reaction Equation (1), which show that anions NO_3_^-^ don’t take part in extracted complex formation. The saling-out effect of nitrate ion is the main reason of increasing of Nd(III) K_d_ values with increasing acidity for SIR 10. The mechanism of Nd(III) recovery by neutral extractant (L) may be presented as:

Nd^3+^ + 3(NO_3_)^−^ + nL = Nd(NO_3_)_3_L_n_ (2)

Thus, the increasing NO_3_^−^ concentration shifts the equilibrium of Reaction (2) to the right. However the salting agent is an electrolyte, and may be solvated by extractant, and can compete with extractad metal for the extractant. That’s why the dependency of Nd(III) K_d_ from HNO_3_ concentration is non-monotonic. To the contrary SIR 11 does not adsorb Nd(III) within the whole examined range of HNO_3_ concentrations as demonstrated in [37]. The influence of HNO_3_ concentration on the value of synergy effect for Nd(III) recovery by SIR 4 is shown in Table 1.

It is noted that values of synergistic effect are much higher in low concentrations of HNO_3_ than in medium ones. A similar result was previously observed when studying the liquid-liquid extraction of REEs with a mixture of 2-(diphenylphosphoryl) methoxy]-5-ethylphenylphosphonate and an ionic liquid [C_4_mim]^+^[Tf_2_N]^−^ [54]. 

#### 2.1.2. The Influence of Component Relation in SIRs

The influence of the L:[C_4_mim]^+^[Tf_2_N]^−^ molar ratio in SIRs on Nd(III) adsorption recovery and values of synergy effect was studied under the same conditions (Table 2). It was found that the capacity of SIRs for Nd(III) rises with an increase of L content in SIRs. The values of Nd(III) K_d_ have the same trend. At the same time the increase of [C_4_mim]^+^[Tf_2_N]^−^ containing in SIRs has the opposite effect, which is probably attributed with fact that excess of ionic liquid in the stationary phase of SIR acts as inert diluent because [C_4_mim]^+^[Tf_2_N]^−^ itself does not extract Nd(III) in all examined range of HNO_3_ concentrations (Figure 2). 

The greatest values of capacities and synergy effects were achieved for SIRs 4 and 5 (Table 2). Since the capacities and synergy effects differ for these SIRs very slightly, a molar ratio of L and [C_4_mim]^+^[Tf_2_N]^−^ of 2:1 was selected for subsequent preparation of SIRs. It should be noted that relatively small amount of [C_4_mim]^+^[Tf_2_N]^−^ needs to be added to L in SIR composition in order to get synergy effect of Nd(III) recovery from slightly acid media (for SIR 6: 0.0291 g [C_4_mim]^+^[Tf_2_N]^−^/1 g SIR (see the experimental section). 

#### 2.1.3. The Influence of the Percentage Extractants

**Figure 3:**
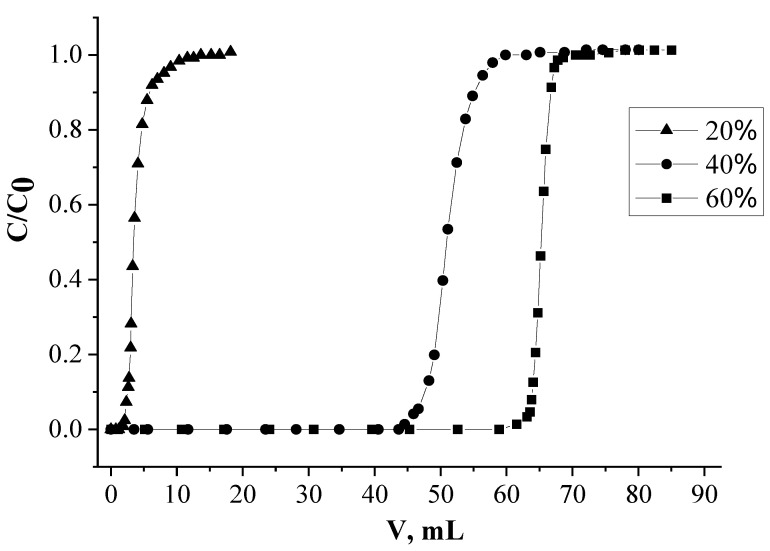
Influence of the extractant percentage in SIRs on recovery of Nd(III).

The percentage of mixture of L and [C_4_mim]^+^[Tf_2_N]^−^ in the SIR influences significantly both the value of Nd(III) distribution coefficient and the synergy effect. The increase of extractant content in SIRs from 20 to 60% was found to raise the value of K_d_ of Nd(III) from 37.4 to 262.4 mL/g, respectively (Figure 3). When the percentage of extractant increases the equilibrium of reaction (Equation (1)) shifts to the right, that is the reason for the Nd(III) K_d_ enhancement. On the other hand the growth of values of Nd(III) K_d_ is limited by the load capacity of SIRs on extractants. Herewith, the maximal value of synergy effect was attained for the SIR containing 60% mixture (L:[C_4_mim]^+^[Tf_2_N]^−^ = 2:1) (SIR 4) (Table 3). 

#### 2.1.4. The Influence of Nd(III) Concentration 

**Figure 4:**
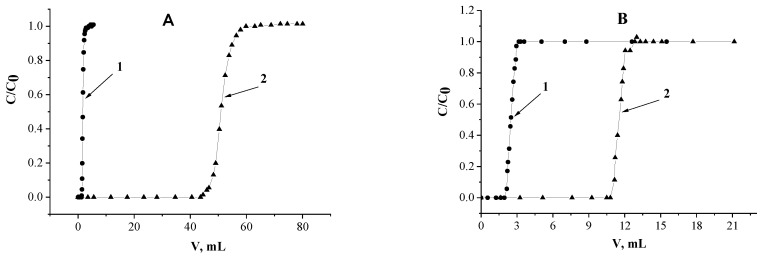
Influence of Nd(III) concentration in the eluent on its adsorption and the values of synergy effect. **1**–SIR 10; **2**–SIR 4. The values of Nd(III) concentration are equal 15 and 150 mg/L for experiments (**A**,**B**), respectively.

Figure 4 illustrates the influence of Nd(III) concentration in eluent on recovery of this metal with SIR impregnated only by L (SIR 10) and SIR 4, which contains the mixture of L and [C_4_mim]^+^[Tf_2_N]^−^ from HNO_3_ of 0.001 mol/L. It was shown that SIR 10 does not adsorb Nd(III) under these conditions. This is due to the fact that phosphorylpodand L is a neutral extractant, and it does not extract metals from slightly acidic media if lacking enough salting-out agent [34]. If the concentration of Nd(III) in the eluent increases from 15 up to 150 mg/L, K_d_ of Nd(III) drops significantly from 209.6 to 47.6 mL/g for SIR 4, which is reason of decrease of synergy effect values (Equation (5)). The values of synergy effect for experiments A and B are equal to 18.6 and 4.25, respectively.

The decrease of K_d_ with the rise of metal concentration in aqueous phase (eluent) is typical for various resins used in liquid chromatography and is attributed to a limited number of adsorption centers on the surface of resins [60]. It should be noted that SIR containing only [C_4_mim]^+^[Tf_2_N]^−^ (SIR 11) does not recover Nd(III) in these conditions, for this reason frontal loading curves for this SIR are not presented on Figure 4.

#### 2.1.5. The Influence of Acid Type

**Figure 5:**
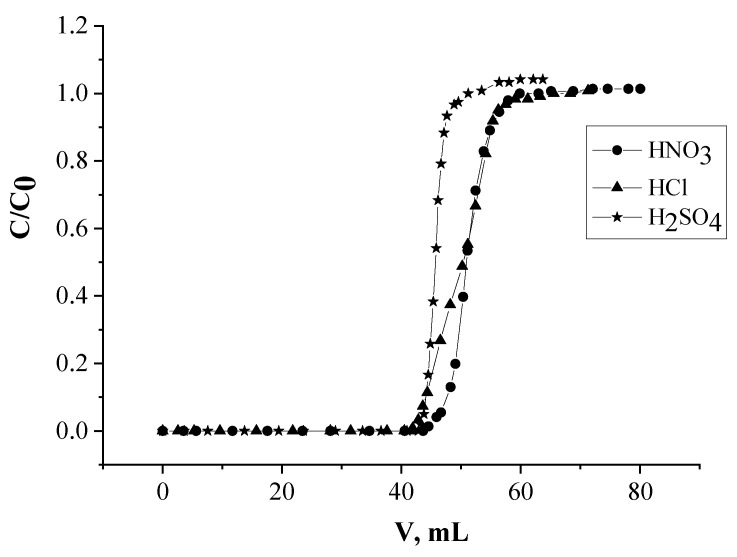
The influence of acid nature on Nd(III) adsorption recovery.

The adsorption recovery of Nd(III) by SIR 4 in nitric, hydrochloric, and sulfuric media was studied. The concentrations of all the chosen acids in the eluent were maintained the same equaling 0.001 mol/L. It was found that replacement of HNO_3_ for HCl does not change values of Nd(III) K_d_ or SIR capacity for the metal (Figure 5). With H_2_SO_4_ the adsorption of Nd(III) is somewhat worse, which is likely due to a higher energy of hydration of sulfate ions [62] and their strong interaction with cations of REEs [63].

Since the values of Nd(III) K_d_ at presence of HNO_3_, HCl, and H_2_SO_4_ differ insignificantly, SIR 4 may be used for REEs recovery from slightly sulfuric medium, which may be important at practical side [64,65,66]. 

### 2.2. Selectivity of REEs Recovery by SIR 4

**Figure 6:**
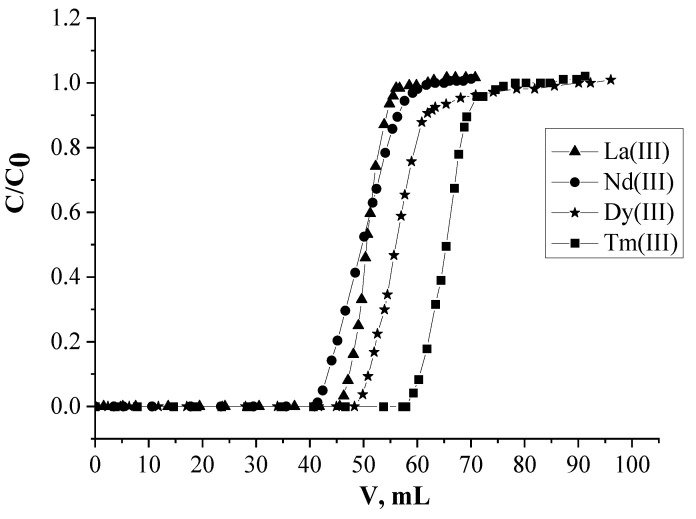
Frontal loading curves of La(III), Nd(III), Dy(III) and Tm(III) in HNO_3_ of 0.01 mol/L.

The adsorptive recovery of La(III), Nd(III), Dy(III), and Tm(III), as representatives of light, medium, and heavy REEs, in HNO_3_ of 0.01 mol/Lby SIR 4 was studied. It is noticed that SIR 10 and SIR 11 containing only L and only [C_4_mim]^+^[Tf_2_N] don’t adsorb these REEs in such conditions (Frontal curves of REEs for these SIRs in Figure 6 are not presented). It means that introduction of [C_4_mim]^+^[Tf_2_N]^−^ into the resin composition results in significant enhancement of recovery efficiency of La(III), Dy(III), and Tm(III) in HNO_3_ of 0.01 mol/L (Figure 6), as in the case of Nd(III). The values of synergy effect of La(III), Nd(III), Dy(III) and Tm(III) recovery by SIR 4 are 15.1, 14.8, 16.7 and 19.4, respectively.

The values of distribution coefficients increase non-monotonously during the transition from La(III) to Tm(III) (Table 4). Probably, this should be attributed to the fact that as the charges of Ln^3+^ ions get denser because of smaller ionic radii with the increase of Z, the complexes of the REEs with L become more stable] [67]. Therefore heavy REEs are adsorbed by SIR 4 much better then light ones. The values of separation factors increase in the trend β_Nd/La_ < β_Dy/Nd_ < β_Tm/Nd_, thus Nd(III) are more easily separated from heavy REEs then light ones.

The values of β is higher for Dy(III)/Nd(III) and Tm(III)/Nd(III) than for Nd(III)/La(III). Thus Nd(III) is more easily separated from heavy REEs than light ones. It occurs because in case of heavy REEs extractad complexes are more stable than light ones, which is probably due to the cation exchanged mechanism of its recovery and the increase of charge density at cross from light REEs to heavy ones.

### 2.3. Extraction Chromatography Recovery of Nd(III) 

**Figure 7:**
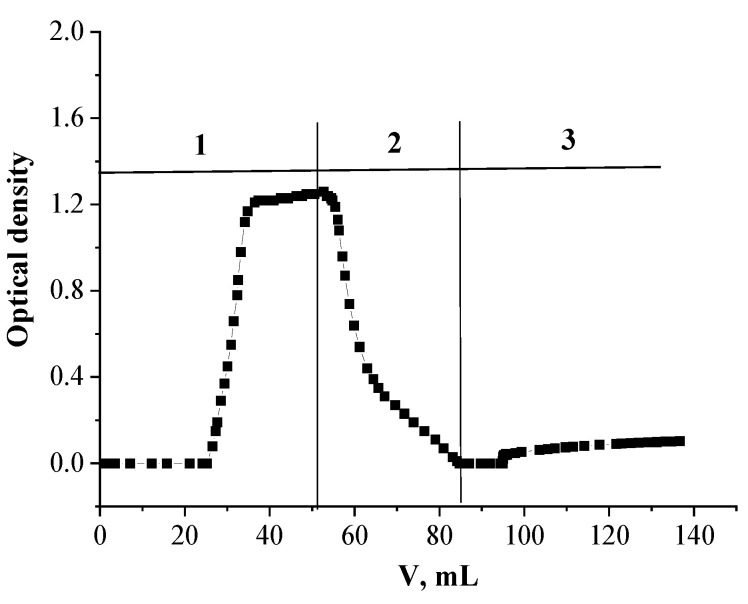
Extraction chromatography recovery of Nd(III) with SIR 4. **1**: adsorption of Nd(III) from HNO_3_ of 0.001 mol/L; **2**: rinse of the column by HNO_3_ with concentration of 0.01 mol/L; **3**: stripping of Nd(III) with distilled water.

Based on performed experiments, the following approach to Nd(III) recovery with SIR 4 was suggested. A feed solution of Nd(III) of a precisely defined concentration of 15 mg/L in HNO_3_ of 0.001 mol/L was continuously put through a column containing SIR 4 until the complete saturation of SIR with Nd(III). Then 45 mL of HNO_3_ solution of 0.001 mol/L were pumped through the column to remove the remaining Nd(III) from the intergrain space. After that Nd(III) was stripped by distilled water. The breakthrough curves for Nd(III) are given in Figure 7.

The capacity of the SIR 4 for Nd(III) before breakthrough and its full load capacity for the metal are 1.31 and 2.27 mg Nd/1 g SIR, respectively (Figure 7). The reason for the low load capacity of Nd(III) for SIR 4, is probably due to the losses of extractant from SIR 4, which are likely conditioned by the small affinity of extractants to the support (LPS-500). Despite of the fact that K_d_ of Nd(III) is minimal in the presence of distilled water (Figure 2), the stripping of Nd(III) with distilled water is too slow for its quantitative recovery. 

**Figure 8:**
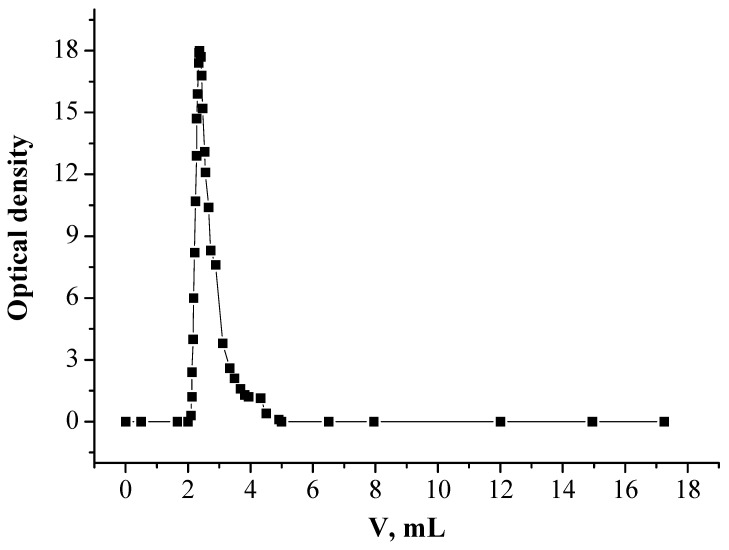
Stripping of Nd(III) with EDTA solution.

In order to improve the Nd(III) stripping, EDTA solution of 0.1 mol/L was used. It was shown earlier in [48] that EDTA is efficient for back-extraction of REEs from organic phase which consists of the mixture of a neutral organophosphorus extractant and ionic liquid. Preliminary SIR 4 was loaded with Nd(III) by passing the solution of Nd(III) with concentration of 15 mg/L in HNO_3_ of 0.001 mol/L through the column, packed with 1.0 g of SIR 4, until full saturation of SIR 4 with Nd(III). Thus in these conditions 2.27 mg of Nd(III) was loaded in SIR 4. The stripping of Nd(III) with EDTA of 0.1 mol/L from SIR 4 is given in Figure 8.

It was found that Nd(III) is stripped quantitatively by putting 6.0 mL of EDTA solution with concentration of 0.1 mol/L through SIR 4. The concentration of Nd(III) in eluate was 376.7 mg/L. Thus the extent stripping of Nd(III) is 99.5% in such condition. Thus, the advantage of SIR 4, at contrast to generally used SIRs, is that recovery and stripping of Nd(III) may be performed from practically neutral solutions which allows to avoid the equipment corrosion.

### 2.4. Comparison of SIRs

**Figure 9:**
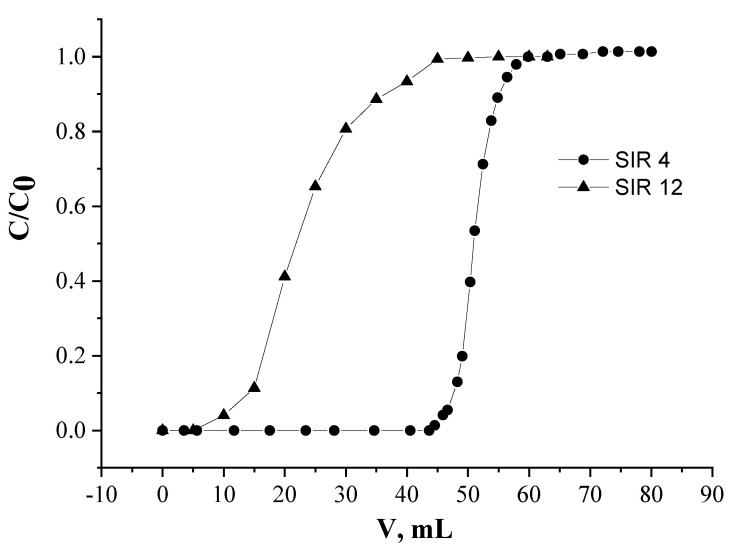
Frontal loading curves of Nd(III) for SIR 4 and SIR 12 in HNO_3_ of 0.001 mol/L.

The closest analog of SIR 4, which is used for REEs recovery is the SIR impregnated by mixture of Cyanex 923 (a mixture of monodentate trialkylphosphine oxides) and ionic liquids [38,68,69]. We prepared SIR 12 containing 40% of mixture Cyanex 923 and [C_4_mim]^+^[Tf_2_N]^−^ in molar ratio of 2:1 and compared efficiency recovery of Nd(III) with SIR 4 in identical conditions (Figure 9). 

The results of this experiment clearly show that recovery of Nd(III) is more effectively performed by SIR 4 compared to SIR 12. The values of full load capacity for the metal are 2.27 and 1.39 mg Nd/1 g SIR for SIRs 4 and 12, respectively. SIR 4, containing polydentate extractant L, turned out to be more effective at Nd(III) recovery, than SIR 12 based on monodentante Cyanex 923, probably, because in the case of L Nd(III) is coordinated by two P=O groups. Previously, we have synthesized the complex of Nd(III) with L and determined its structure by X-ray diffraction. The comprehensive tables of the atomic coordinates, bond lengths, and bond angles in structures L, and its complex with Nd(III) have been deposited with the Cambridge Crystallographic Data Centre (nos. 907137–97139; deposit@ccdc.cam.ac.uk (accessed on 20 April 2021) or http://www.ccdc.cam.ac.uk/data_request/cif (accessed on 20 April 2021)). An analysis of the obtained results allowed us to conclude that the binding of the neodymium ion by the extractant L occurs due to the coordination with four phosphoryl groups of two L molecules, which was further confirmed by IR spectroscopy [35].

## 3. Experimental Section

### 3.1. Synthesis

The structure and the purity degree of synthesized compound L was ascertained with NMR spectroscopy and elemental analysis data. The content of C, H was established with standard methods using a Carlo Erba CHN analyzer (Erba Group, Brno, Czech Republic). NMR spectra were registered with a CXP-200 or Bruker-DXP-200 (200 MHz) instrument (Bruker, MA, USA) with tetramethylsilane as the internal standard, while for ^31^P NMR 85% H_3_PO_4_ was used as reference. The control of composition of reaction mixture was conducted by the method of thin-layer chromatography on Silufol plates (Merck, NJ, USA). The mixture chloroform:isopropanol = 10:1 was used as eluent. The development of chromatograms was performed by fuming iodine.

**((2-Diphenylphosphoryl)-4-ethylphenoxy)methyl)diphenylphosphine oxide** (**L**). A mixture of 5.0 g (15.5 mmoL) (5-ethyl-2-hydroxyphenyl)diphenylphosphine oxide, 6.00 g (15.5 mmoL) diphenylphosphoryl)methylbebzenesulfonate and 5.1 g (15.5 mmoL) anhydrous cesium carbonate in 45 mL dioxane was heated and stirred at 100 °C for 10 h. The reaction mixture was diluted by 50 mL of water, acidified by adding concentrated HCl to pH 1, and extracted by CHCl_3_ (3 × 25 mL). The organic layer was separated, washed with water, and evaporated under reduced pressure to give 7.25 g (86%) of crude product. After recrystallization from a benzene-hexane mixture (1:1), 6.6 g (79%) of compound L was obtained, mp = 166–168 °C (The melting temperature is established with a short Anschutz thermometer). It was found, %: C 73.63, 73.50; H 5.35, 5.60; P 11.49, 11.39. For C_33_H_30_O_3_P_2_. It was calculated, %: C 73.87; H 5.64; P 11.55. ^1^H-NMR δ, ppm (CDCl_3_): 1.12 t (6H, ^3^*J*_H_H_ = 7.02 Hz CH_3_CH_2__Ar), 2.55 q (2H, ^3^*J*_H-H_ = 7.58 Hz CH_3_CH_2_Ar), 4.49 d (2H, ^2^*J*_H-P_ = 5.18 Hz OCH_2_P(O)Ph_2_), 7.07 m (1H, Ar-H), 7.30–7.65 m (22H, Ar–H). ^31^P-NMR, δ, ppm (CDCl_3_): 28.13, 29.92.

Bis[(trifluoromethyl)sulfonyl]imide-1-buthyl-3-methylimidazolium was provided by Sorbent-Technologies, Ltd. (Moscow, Russia). This compound was “purim” grade and assay an ≥99% (GC). The purity of synthesized compounds was ≥99.0% (according to the NMR data and elemental analysis results). 

### 3.2. Preparation of Solutions and Analysis

**Figure 10:**
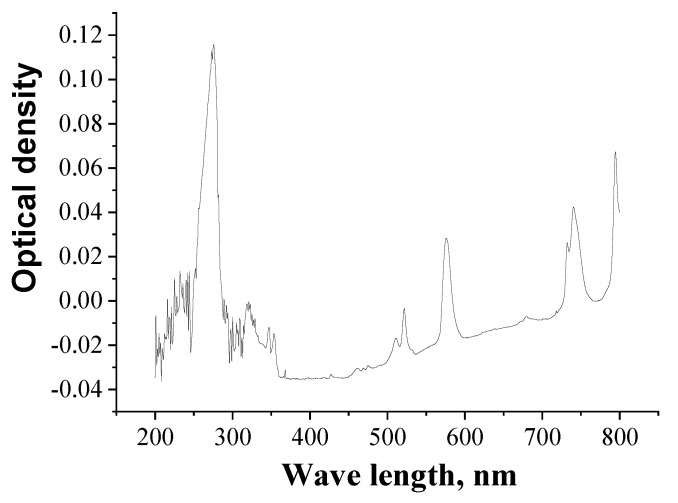
Specific absorption spectrum of Nd(III) solution in HNO_3_ of 1 mol/L.

Nitric acid solutions of Nd(III) were prepared by dissolving precisely weighed portions of Nd_2_O_3_ (purity > 99.9%, Aldrich, Germany)in nitric acid solutions of the corresponding concentrations. Nitric acid solutions were prepared by diluting concentrated HNO_3_. The concentrations of the obtained diluted solutions of HNO_3_ were determined by titration with the standard solution of NaOH in the presence of bromothymol blue. Solutions of Arsenazo M (ACS Reagent grade, Acros Organics, Belgium were prepared by dissolving precisely weighed portions of the reagent in distilled water. Solutions of EDTA (“purum” grade, Acros Organics) were prepared similarly. The concentrations of Nd(III) in the eluates were evaluated spectrophotometrically using Arsenazo M] [70]. All reagents used were analytical grade. The measurements of optical density of Nd(III) solutions flowing from the column were performed automatically with a spectrophotometric detector and software. With concentration of HNO_3_ in eluates exceeding 0.5 mol/L, the concentration of Nd(III) in such solutions was detected spectrophotometrically using specific absorption spectrum of Nd(III) (Figure 10), with the wavelength of 576 nm] [71]. 

### 3.3. Preparation of SIRs

The studied SIRs (Table 5) were prepared with method described earlier in [38]. Weighed portions of L and ionic liquid [C_4_mim]^+^[Tf_2_N]^−^ (Table 6) were dissolved in 30 mL CHCl_3_ and mixed with the suspension of copolymer of styrene with divinylbenzene LPS-500 (specific surface area is 570 m^2^/g, diameter of pores is 3–50 μm, size of particles is 40–70 μm) (provided by RossPolimer, Moscow, Russia) in approx. 20 mL CHCl_3_. The resulting mixture was stirred in the rotating flask of the rotary evaporator, and then CHCl_3_ was removed with vacuum at 50 °C. Having collected all the condensate and not seeing bubbles in the suspension, the SIR was stirred in complete vacuum at 40–50 °C for 30 min for full removal of CHCl_3_. 

### 3.4. Equipment

The recovery of Nd(III) was studied in dynamic mode on an automatic chromatographic device manufactured by Knauer (Germany), consisting of three high pressure pumps, dosing valve, chromatography column, and a spectrophotometric detector. The recovery of Nd(III) was carried out using a plastic column with the length of 100 mm and the internal diameter equaling 4 mm, respectively. The column was packed with resins by the “dry method”, loading dry resin inside the column in small portions and compacting it by a glass rod. The physical constants of the prepared columns are presented in Table 7.

### 3.5. Batch Uptake of Nd(III)

**Figure 11:**
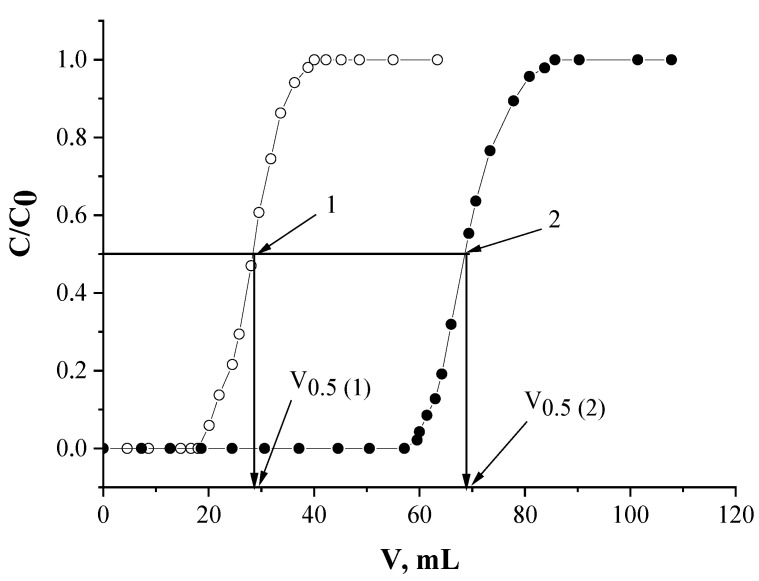
The approach of values calculation of metal distribution coefficients using frontal loading curves.

The chromatographic column stuffed with SIRs (Table 1) was rinsed using a peristaltic pump by HNO_3_ solution of a chosen concentration with a flow rate of 1 mL/min for 1 h. Then Nd(III) solution of a certain concentration, which was previously determined by titration with standard solution of EDTA] [72], in HNO_3_ of the same concentration as on the rinsing step was constantly passed through the column until the complete SIR saturation with a flow rate of 0.5 mL/min. The Nd(III) concentrations in eluates which left the column were automatically determined by the spectrophotometric method. The obtained frontal loading curves (Figure 11) were used to calculate the values of distribution coefficients and the capacity of the SIRs.

The dynamic distribution coefficients (K_d_, mL/g) were calculated per Equation (3)] [60]:

K_d_ = V_0.5_/m_e_, (3)

where V_0.5_ is the volume of solution until half breakthrough of metal, mL; m_e_ is the mass of extractant in the resin, g.

The values of separation factors (β) were calculated per Equation (4):

β = K_d2_/K_d1,_ (4)

where K_d1_ and K_d2_ are distribution coefficients of separating metals

The values of the synergy effect (SE) were calculated per Equation (4):

SE = K_d(L+IL)_/K_d L_+K_d IL_ (5)

where K_d(L+IL)_, K_d L_ and K_d IL_ (mg/L) are values of the distribution coefficient obtained for SIRs impregnated by mixture of phosphorylpodand L with ionic liquid, phosphorylpodand L alone and ionic liquid alone, respectively.

## 4. Conclusions

Novel solvent impregnated resins (SIRs) containing the mixture of the promising polydentante extractant (2-diphenylphosphoryl)-4-ethylphenoxy)methyl)diphenyl-phosphine oxide (L) and the ionic liquid [C_4_mim]^+^[Tf_2_N]^−^ were prepared for adsorptive recovery of Nd(III). The performed study was dealt with investigation of influence of [C_4_mim]^+^[Tf_2_N]^−^ on the recovery efficiency of Nd(III) using these resins. It was shown that the addition of even a small amount of ionic liquid (0.0291 g [C_4_mim]^+^[Tf_2_N]^−^/1 g resin) to phosphorylpodand **L** into the SIR composition results in significant enhancement of Nd(III) recovery from slightly acidic nitric solutions. The values of synergy effect are maximal with the concentration of HNO_3_ from 0.001 to 0.1 mol/L, are close to 1.0 in the range from 0.5 to 1.0 mol/L, and for the concentrations of HNO_3_ over 1 mol/L there is no synergy effect. It was established that the synergy effect is proportional to the amount of **L** in the SIRs and inversely proportional to the amount of [C_4_mim]^+^[Tf_2_N]^−^. The percentage of extractants in SIRs was optimized. The most efficient SIR for Nd(III) recovery is that containing 40% mixture of L with [C_4_mim]^+^[Tf_2_N]^−^ in a molar ratio of 2:1 (SIR 4). The optimal conditions for adsorption and stripping of Nd(III) with this SIR were chosen. Nd(III) is most efficiently adsorbed from HNO_3_ with concentration of 0.01 mol/L. In these conditions 1.31 mg Nd(III)/1 g of SIR 4 can be loaded without breakthrough and then can be stripped quantitatively by putting 6.0 mL of EDTA solution with concentration of 0.1 mol/L through the column. The selectivity of Nd(III) recovery was studied. It was found that Nd(III) is easier to separate from heavy REEs then light ones. It was found that in recovery efficiency of Nd(III) novel SIR exceeded the SIR containing mixture of Cyanex 923 and [C_4_mim]^+^[Tf_2_N]^−^. The results of this work showed that Nd(III) adsorption with SIRs impregnated by **L** is improved significantly by introducing [C_4_mim]^+^[Tf_2_N]^−^ into the SIR composition, and it can be used for the recovery of Nd(III) from slightly acidic media. 

## Figures and Tables

**Table 1:** The values of synergy effect (SE) of Nd(III) recovery by SIR 4 for various concentrations of HNO_3_.

Concentration of HNO_3_, mol/L	SE = K_d(L+IL)_/K_d L_+K_d IL_
0	1.60
0.001	15.1
0.01	16.5
0.1	3.2
0.5	1.23
1.0	0.79
3.0	0.39

**Table 2:** The values of distribution coefficient (K_d_), capacity and synergy effect (SE) obtained for SIRs of different composition in Nd(III) recovery from HNO_3_ of 0.001 mol/L.

SIR *	Molar Ratio L:[C_4_mim]^+^[Tf_2_N]^−^	K_d,_ mL/g	Capacity, mg Nd/1 g Resin	SE
1	1:1	222	1.52	12.7
2	1:2	184	1.19	10.6
3	1:4	131	0.98	7.53
4	2:1	260	2.0	14.9
5	5:1	274	2.04	15.7
6	10:1	226	2.62	13.0

* All SIRs contain 40% extractants.

**Table 3:** The values of synergy effect (SE) for recovery of Nd(III) from HNO_3_ of 0.001 mol/L by SIRs containing the mixture (L:[C_4_mim]^+^[Tf_2_N]^−^ = 2:1).

Content of Extractants, %	SE
20	1.08
40	12.9
60	15.1

**Table 4:** The values of distribution coefficients (K_d_) and separation factors (β_REE/Nd_) for La(III), Nd(III), Dy(III), and Tm(III).

Rare Earth Element	K_d_, mL/g	β_REE_/_Nd_
La(III)	242.8	1.03
Nd(III)	250.5	_
Dy(III)	289.2	1.15
Tm(III)	325.4	1.30

**Table 5:** Composition of prepared SIRs.

SIR	Composition of Stationary Phase (Mole Ratio of L and [C_4_mim]^+^[Tf_2_N]^−^	Extractant Content, %
1	1:1	40
2	1:2	40
3	1:4	40
4	2:1	40
5	5:1	40
6	10:1	40
7	2:1	20
8	2:1	60
9	2:1	75
10	L	40
11	[C_4_mim]^+^[Tf_2_N]^−^	40
12	Cyanex 923:[C_4_mim]^+^[Tf_2_N] = 2:1	40

**Table 6:** Weighed portions of substances used to prepare SIRs *.

SIR *	Weight of Portion, g		
	L	[C_4_mim]^+^[Tf_2_N]^−^	LPS-500
1	0.2241	0.1755	0.6001
2	0.1556	0.2444	0.6000
3	0.0965	0.3033	0.6002
4	0.2870	0.1128	0.6002
5	0.3455	0.0534	0.6003
6	0.3707	0.0291	0.6000
7	0.1436	0.0564	0.8001
8	0.4311	0.1691	0.4003
9	0.5387	0.2115	0.2502
10	0.4001	-	0.6002
11	-	0.4003	0.6001

* Per 1 g of SIR. For preparing 1 g of SIR 12 we need 0.2590 g of Cyanex 923 and 0.1411 g [C_4_mim]^+^[Tf_2_N]^−^.

**Table 7:** Physical constants of columns packed with developed SIRs.

SIR	Extractant Density, (g/mL)	Bed Density (g/mL)	V_s_, mL	V_m_, mL	V_s_/V_m_
1	1.40	1.18	0.27	1.14	0.24
2	1.39	1.17	0.27	1.12	0.24
3	1.41	1.19	0.27	1.14	0.24
4	1.42	1.18	0.27	1.18	0.23
5	1.44	1.20	0.28	1.55	0.18
6	1.46	1.23	0.28	1.43	0.20
7	1.42	1.21	0.41	0.80	0.51
8	1.42	1.22	0.51	0.25	2.04
9	1.40	1.18	0.27	0.84	0.32
10	1.46	1.24	0.28	1.58	0.18
11	1.38	1.16	0.67	0.59	1.13
12	1.40	1.18	0.65	0.63	1.03

Vs is the volume of extractant, which is held by SIR; Vm is the volume of eluent, which is located inside column packed with SIR.

## Data Availability

Not available.
